# Cell line-specific efficacy of thermoradiotherapy in human and canine cancer cells *in vitro*

**DOI:** 10.1371/journal.pone.0216744

**Published:** 2019-05-15

**Authors:** Katarzyna J. Nytko, Pauline Thumser-Henner, Mathias S. Weyland, Stephan Scheidegger, Carla Rohrer Bley

**Affiliations:** 1 Division of Radiation Oncology, Vetsuisse Faculty University of Zurich, Zurich, Switzerland; 2 Center for Applied Biotechnology and Molecular Medicine, University of Zurich Zurich, Switzerland; 3 Center for Clinical Studies at the Vetsuisse Faculty of the University of Zurich, Zurich, Switzerland; 4 ZHAW School of Engineering, Zurich University of Applied Sciences, Winterthur, Switzerland; 5 BioNanomaterials Group, Adolphe Merkle Institute, University of Fribourg, Fribourg, Switzerland; Colorado State University, UNITED STATES

## Abstract

**Objective:**

Aims were to investigate sensitivity of various human and canine cancer cell lines to hyperthermia and the influence of particular treatment conditions, and to analyze the DNA-damage response and mode of cell death in cell line radiosensitized by hyperthermia. Additionally, we were interested in the involvement of HSP70 in radiosensitization.

**Methods:**

Radiosensitization by hyperthermia was determined in a panel of human and canine cancer cell lines using clonogenic cell survival assay, as well as levels of heat shock proteins (HSPs) using immunoblotting. The influence of the hyperthermia-radiotherapy time gap, different temperatures and the order of treatments on clonogenicity of hyperthermia-sensitive A549 cells was investigated. Additionally, DNA damage and cell death were assessed by Comet assay and an apoptosis/necrosis assay. Further we induced transient knockdown in A549 cells to test HSP70’s involvement in radiosensitization.

**Results:**

Out of eight cell lines tested, only two (A549 and Abrams) showed significant decrease in clonogenic cell survival when pre-treated with hyperthermia at 42°C. Strong induction of HSP70 upon thermoradiotherapy (HT-RT) treatment was found in all cell lines. Transient knockdown of HSP70 in A549 cells did not result in decrease of clonogenic cell survival in response to HT-RT.

**Conclusion:**

Tumor cell-type, temperature and order of treatment play an important role in radiosensitization by hyperthermia. However, hyperthermia has limited potency to radiosensitize canine cancer cells grown in a 2D cell culture setting presented here. DNA damage and apoptosis/necrosis did not increase upon combined treatment and cytosolic levels of HSP70 appear not to play critical role in the radiosensitization of A549 cells.

## Introduction

Radiotherapy remains one of the major treatment options in human and animal cancer treatment. Unfortunately, due to the intrinsic resistance, many solid tumors are radiation-resistant. Tumor hypoxia, DNA damage repair capacity and tumor microenvironment are the major determinants of sensitivity towards radiotherapy.

Pre-treatment of cells with hyperthermia (40–43°C) can be used to sensitize tumor tissues to the subsequent radiotherapy treatment; this concept was described decades ago [1, 2]. The mechanism of radiosensitization by hyperthermia is multifold and depends on many parameters such as the tumor type or levels of tumor hypoxia. Hyperthermia induces the cellular and tumor microenvironment changes, which can alter the response to radiotherapy. Acting on both, tumor microenvironment and cellular level, hyperthermia has been shown to reduce tumor hypoxia by increasing perfusion [3]. The effects of hyperthermia on tumor perfusion and oxygenation status have been well characterized [4]. On the other hand, the direct effects of thermoradiotherapy (perfusion- and hypoxia-independent) on tumor cells alone are yet to be fully elucidated. Both, the cellular and microenvironment-related effects of hyperthermia are mediated, among others factors, by heat shock proteins (HSPs). HSPs are molecular chaperones induced in response to stresses such as heat, their major function is to help the cell to adapt to stress conditions and to properly respond to the second stress insult [5]. There are several members of the heat shock protein family including HSP27, HSP70 and HSP90 being the best characterized. Their protein levels have been shown to be induced in many malignancies, such as prostate, colorectal carcinoma and ovarian cancer [6]. The role of HSPs proteins in radio-modulating the effect of hyperthermia is multifold. On one hand, they contribute to treatment resistance by helping the cell to adapt to stress conditions and on the other hand they contribute to the immune response to the tumor, which can be complementary to radiotherapy treatment [7]. Inhibitors of HSP70 and HSP90 have been reported to have antiproliferative and cytotoxic effect on different cancer cell types, including canine osteosarcoma [8]. Moreover, it has been shown that the knockout of HSP70 (HSP70.1 and HSP70.3, mouse HSP70) in mice resulted in genomic instability, suggesting that HSP70 might play a role in the DNA damage response, which is one of the main factors responsible for the response to radiotherapy [9]. Inhibition of HSP70 expression by siRNA has been shown to be cytotoxic in different types of tumor but not in normal tissue [6].

The aim of our study was twofold. First, to screen the human and canine cancer cell lines for their sensitivity towards hyperthermia-radiotherapy treatment using clonogenic cell survival assay as a read-out and to analyze the effect of thermoradiotherapy on DNA damage, and apoptosis/necrosis [10]. Second, to investigate the role of HSP70 protein in mechanism of the radiotherapy-sensitization by hyperthermia, we compared the levels of HSP70 induction in hyperthermia-sensitive and–resistant cell lines. The aim was to investigate, whether levels of basal and inducible HSP70 could correlate with response rate to thermoradiotherapy *in vitro*. Additionally, using the cancer cell lines grown without presence of any additional cell type such as endothelial and immune cells (hence, excluding the effect of tumor microenvironment), we wanted to investigate the effect of hyperthermia treatment on cancer cells alone, in particular the effects on DNA damage and apoptosis/necrosis. In this context, we could study the perfusion- and hypoxia-independent effects of hyperthermia treatment on cancer cells. Moreover, little is known about response of the canine cancer cell lines to thermoradiotherapy and how they compare to the human cancer. In particular, we focused canine sarcoma cell lines, since inoperable or recurrent sarcoma is one of the disease entities for treatment with hyperthermia in veterinary medicine [11, 12].

In our study, we used clonogenic cell survival assay and proliferation assay to assess the efficacy of the combined thermoradiotherapy treatment, Comet assay to investigate the DNA damage, apoptosis/necrosis assay to investigate the cell death, and we analyzed the HSP70 after combined treatment by immunoblotting.

## Material and methods

### Cell lines

Human lung adenocarcinoma cell line A549 was a kind gift of Prof. Martin Pruschy (University Hospital Zurich, Zürich, Switzerland). Canine soft tissues sarcoma (K9 STS) was a kind gift of Prof. Marlene Hauck (NC State University, North Carolina, USA). Canine osteosarcoma (UCDK9OSA17, Abrams, D17) and melanoma (UCDK9MM2) were kind gifts of Prof. Robert Rebhun (University of California, Davis, California, USA). Canine transitional cell carcinoma (K9 TCC) was a kind gift of Prof. Deborah Knapp (Purdue University, Indiana, USA). Human osteosarcoma U2OS was a kind gift of Prof. Michael Hottiger (University of Zürich, Zürich, Switzerland). A549 and K9 STS cells were maintained in RPMI 1640 cell culture media medium supplemented with 10% FBS (Gibco), 1x GlutaMAX (Gibco), penicillin-streptomycin (100 U/ml-100 μg/ml, Gibco), 1x non-essential amino-acids (Gibco), 1 mM sodium pyruvate (Sigma) and 10 mM HEPES (Gibco). UCDK9OSA17, Abrams, D17, U20S, K9 TCC and UCDK9MM2 were maintained in DMEM high glucose/pyruvate/L-glutamine cell culture media (Invitrogen) supplemented with 10% FBS (Gibco), penicillin-streptomycin (100 U/ml-100 μg/ml, Gibco) and 10 mM HEPES (Gibco). All cell lines used in the study were tested mycoplasma-free.

### Heating method and ionizing radiation

**Fig 1:**
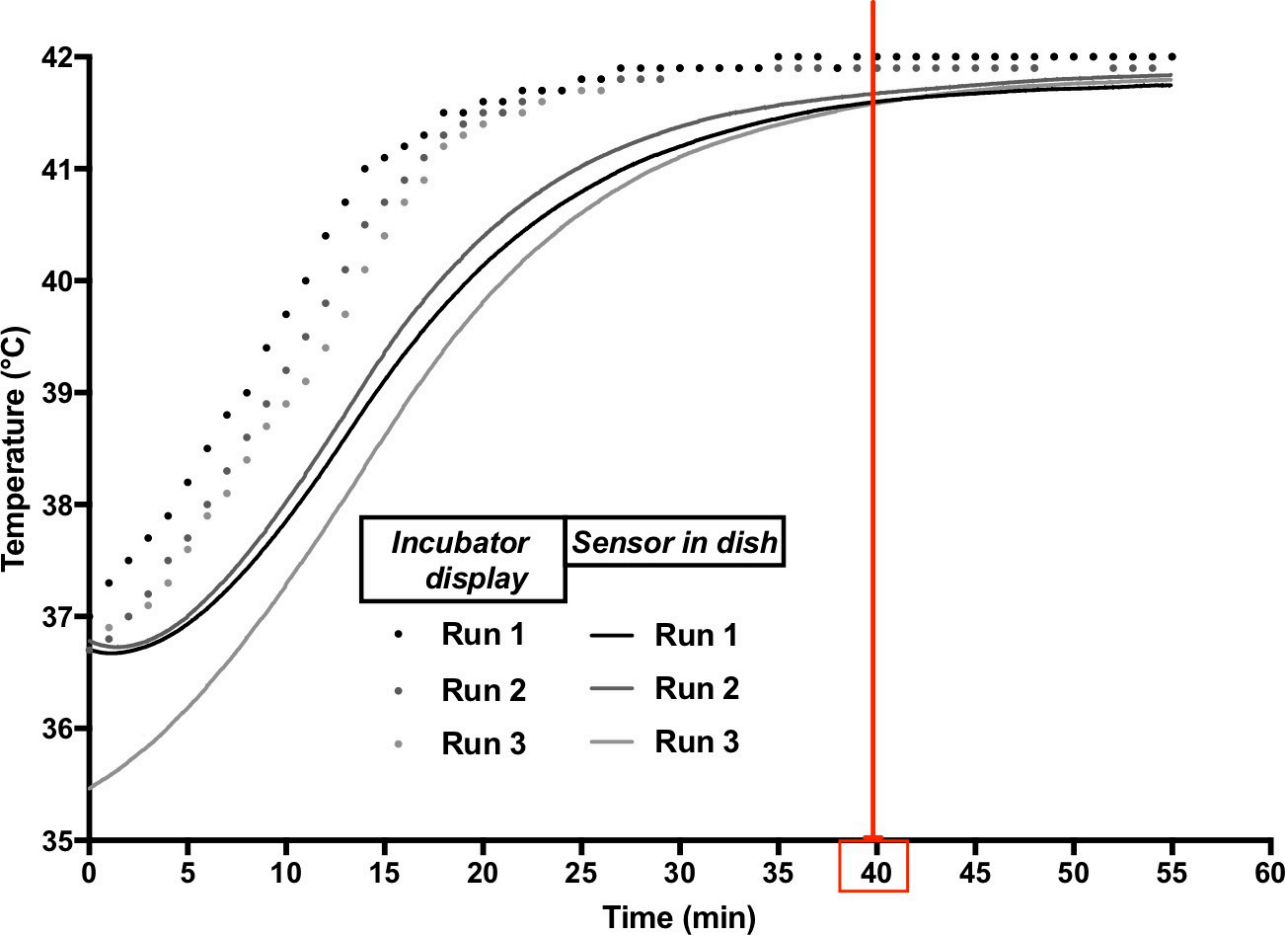
Temperature profile of the incubator during heat-up phase to 42°C steady-state hyperthermia. Dots represent temperature read off the incubator display over three independent runs, and lines represent the temperature recorded by a sensor placed in the culture dish, immersed in medium during those same runs.

Control cells were maintained at 37°C in a humidified incubator with 5% CO_2_. Cells treated with hyperthermia were incubated in the same type of humidified incubator from the start of the heat-up phase, which took approximately 40 minutes. The subsequent hyperthermia treatment at the target temperature (42°C) took another hour. Afterwards cells were removed from the hyperthermia incubator and irradiated with 3 or 6 Gy (or as indicated in the figure legend). In order to ensure that the cells are exposed to a repeatable temperature profile similar to what we observe during *in vivo* treatments [12], we ran a series of thermometry measurements on our incubator setup before starting with the treatment of cells. The temperature measured directly (with a Bowman probe (SPEAG/IT’IS, Zurich, Switzerland)) in the cell culture dish is provided in Fig 1 for three independent runs, along with the temperature shown on the incubator’s own temperature display. While the latter typically reaches the target temperature within 25 minutes, the temperature in the dish is within 0.5°C of the target temperature after less than 40 minutes. The incubator’s specifications with respect to Uniformity and Temperature Control Fluctuation are ±0.25°C and ±0.1°C, respectively.

Additionally, the time gap between hyperthermia and irradiation was between 10–20 minutes unless specified otherwise. Irradiation was performed with 6 MV linear accelerator (Clinac iX, Varian, Palo Alto, USA), a field size of 30 × 30 cm, source-surface distance (SSD) of 100 cm, with a dose rate of 600 MU/min (= approximately 6 Gy/min). Control cells were mock-irradiated.

### Clonogenic cell survival assay

Clonogenic cell survival was determined in the cells treated with radiotherapy alone or combined thermoradiotherapy. Cells were seeded into 10 cm petri-dishes the day before treatment start (the cell number to reach optimal (separate single clones) number of clones was optimized for every cell line and every condition beforehand: 0Gy/37°C/42°C—200 cells (A549, Abrams, K9, TCC, MM2, OSA17), 250 cells (U2OS); 2Gy/37°C/42°C—250 cells (K9, MM2, OSA17); 3Gy/37°C/42°C—400 cells (A549, TCC), 250 cells (Abrams) 1000 cells (U2OS); 4Gy/37°C/42°C—500 cells (K9, MM2, OSA17); 6Gy/37°C/42–8000 cells (A549), 4000 cells (U2OS), 500 cells (Abrams), 1000 cells (K9, MM2, OSA17), 800 cells (TCC)). The following day, cells were treated either with hyperthermia (42°C, heat-up plus 1 h) or control (37°C) and ionizing radiation. After colony formation (depending on cell lines, approximately 10–14 days), colonies were fixed (methanol/acetic acid; 3:1) and stained with crystal violet (1%, Sigma). Colonies containing > 50 cells were counted manually using a colony counter. Cell-surviving fraction was calculated by dividing the number of obtained colonies after treatment by the number of seeded cells and correcting for plating efficiency of control cells.

### Proliferation assay

The proliferative activity of tumor cells (A549) was assessed using Cell Counting kit-8 according to the manufacturer protocol (Dojindo Molecular Technologies). Briefly, A549 cells were seeded in a 96-well plate the day before treatment start (500 cells/well). The following day cells were treated with hyperthermia (42°C, heat-up plus 1 h) followed by ionizing radiation (6 Gy) alone or combined treatment. The proliferation (OD at 450 and 600 nm) was measured 24, 48 and 72 hours after irradiation.

### SDS-PAGE and immunoblotting

Cells were seeded (250’000–500’000) in a 10 cm dish the day before experiment start. The following day, cells were treated with hyperthermia (42°C, heat-up plus 1 h) followed by ionizing radiation (6 Gy), protein lysates were collected 2 h after irradiation. Cells were lysed in RIPA buffer (Sigma) with Protease Inhibitor Cocktail (Sigma). The protein concentration was adjusted using BCA assay (Thermo Fisher Scientific). The samples were separated on a 4–15% gradient gel (Bio-Rad) and blotted on PVDF membrane using a transfer apparatus according to manufacturer’s protocol (Bio-Rad). After blocking with 5% nonfat milk in TBST (Bio-Rad), membranes were probed with the following antibodies: anti-HSP70 (C92F3A-5, 1:1000, Santa Cruz and D69, 1:1000, Cell Signaling Technology (S2 Fig)), anti-Rad51 (D4B10, 1:1000, Cell Signalling Technology), anti-HSP90 (ab13492, 1:1000, Abcam) anti beta-actin (8226, 1:1000, Abcam); and secondary anti-mouse IgG, HRP-linked Antibody (#7076, 1:2000, Cell Signaling Technology), and anti-rabbit IgG, HRP-linked Antibody (#7074, 1:1000, Cell Signaling Technology). The proteins were visualized using Pierce ECL Western Blotting Substrate (Thermo Fisher Scientific) and exposed to x-ray film. The quantification of relative protein levels was performed using Fiji software.

### Transient transfection with siRNA

A549 cells were seeded in a 6-well plate at density of 100’000 cells/well the day before transfection. siRNA targeting HSP70 (sc-29352) was purchased from Santa Cruz Biotechnology. Control siRNA was purchased from Microsynth AG using the sequence described before [13]. Transfection with target and control siRNA (25 nM) was performed using TransIT-X2 Dynamic Delivery System (Mirus) according to the manufacturer protocol. Knockdown efficiency was analyzed 48 h after transfection. To test the functional effect of HSP70 knockdown on radiation sensitivity using clonogenic cell survival assay, cells were seeded for clonogenic assay 24 h after transfection and treated with hyperthermia/radiation (as described above) 48 h after transfection.

### Comet assay

A549 cells (150’000 cells/well) were seeded in a 6-well plate the day before experiment start. The following day, cells were treated with hyperthermia (heat-up plus 1 h, 42°C) or control and irradiated with 8 Gy directly after hyperthermia treatment was finished. The cells were trypsinized, counted and resuspended in PBS at a density of 200’000/ml. Afterwards 7.5 μL of cell suspension were mixed with 65 μL of 1% low melting point agarose (LMPA, Trevigen, Gaithersburg, MA, USA) in PBS at 37°C.

Subsequently, 40 μL of agarose-cell solution was pipetted onto 2-well slides (Trevigen, Gaithersburg, MA, USA). The slides were then placed at 4°C for 10 min to allow agarose polymerization. Subsequently, the slides were placed in lysis buffer (Trevigen, Gaithersburg, MA, USA) for 90 min at 4°C in the dark. The slides were then incubated in the alkaline electrophoresis solution (200 mM NaOH; 1 mM EDTA, pH 8.0) for 10 min at 4°C in the dark. The electrophoresis was run at 300 mA for 30 min in an alkaline electrophoresis solution (200 mM NaOH; 1 mM EDTA, pH 8.0). After electrophoresis, slides were washed twice with excess H_2_O for 10 min at room temperature and incubated in 70% ethanol for 15 min at room temperature. After drying (overnight), slides were stained in SybrGold solution 1:10’000 in TE buffer, pH 8.0 for 15 min at room temperature, protected from light. The slides were washed twice with H_2_O at room temperature and dried overnight. The COMET IV scoring system was used to quantify the tail intensity (%DNA in tail) [14].

### Apoptosis and necrosis assay

A549 cells were seeded in a white 96-well plate at a density of 5000/well. The following day, cells were treated with hyperthermia (heat-up plus 1 h, 42°C) alone, radiation (6 Gy) alone or combined thermoradiotherapy. 48 hours after treatment, cells were analyzed with RealTime-Glo Annexin V Apoptosis and Necrosis Assay (Promega) multiplexed with CellTiter-Glo 2.0 Cell Viability Assay (Promega) to normalize for viable cell number according to the manufacturer’s protocol.

### Statistics

Statistical analyses were performed using R (The R Foundation for Statistical Computing, version 3.5.0, 2018); the multicomp package (version 1.4–8) was used to conduct post-hoc tests. To compare two groups two-tailed unpaired Student t-test was performed. Clonogenic cell survival data was analyzed with ANOVA followed by Dunett’s Multiple Comparison test to compare treatment conditions to control. Linear-Quadratic (LQ) predictions were obtained by fitting the equation log_10_(*S*) = - α*D*- β*D*^2^, α, β ≥ 0, where *S* represents the survival fraction and *D* represents the dose. Enhancement factors (EF) of the α and β parameter estimates were calculated by dividing the parameter estimates of the treatment (e.g. treatment with HT as radiosensitizer) by the parameter estimates of the control (e.g. treatment with ionizing radiation, but no HT). The remaining results were analyzed using mixed model ANOVA; a random factor was used to model variation between experimental repetitions and Tukey’s Multiple Comparison Test was used to compare different groups to each other. P values are indicated in the figures.

## Results

### Efficacy of thermoradiotherapy treatment in human and canine cancer cell lines

**Fig 2:**
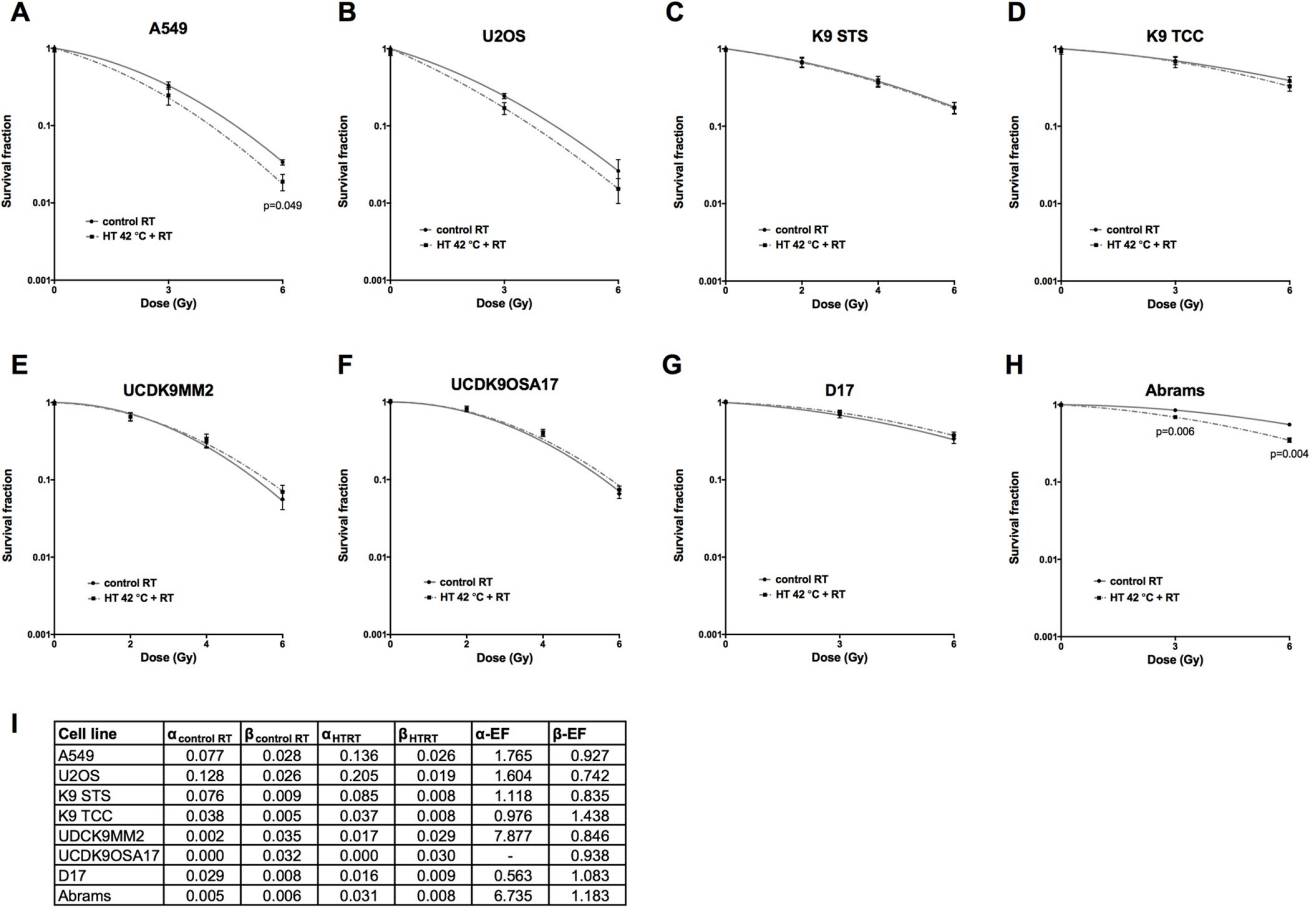
Effect of thermoradiotherapy treatment on clonogenic cell survival of cancer cells. Clonogenic cell survival curves of human (A, B) and canine (C-H) cancer cell lines. The corresponding LQ parameters along with their enhancement factors are given in a Table (I). Unpaired t-test. Mean of at least three independent experiments ±SEM is shown.

We tested the effect of hyperthermia in the panel of human and canine cancer cell lines of different origin using clonogenic cell survival assay. While human A549 lung adenocarcinoma cells and canine Abrams osteosarcoma cells were radiosensitized by pre-treatment with hyperthermia, all other cell lines did not show radiosensitization (Fig 2A–2H).

According to prior research, hyperthermia should increase the α parameter of LQ model by an EF of 2 or even more while the β should remain relatively stable, i.e. β-EF ≈ 1[15]. For the two cell lines subject to radiosensitization, this was the case. The U2OS also had an elevated α-EF (1.6). Indeed, while not statistically significant, a radiosensitization effect is visible in the plot. The large α-EF (7.9) of the UDCK9MM2 cell line is an artifact of very small α estimates.

Moreover, we tested if the single and combined treatments have an effect on A549 cells proliferation over a 72 h period after treatment. Interestingly, hyperthermia alone treated cells were proliferating faster than control at 24h time point but at later time points their proliferation was significantly reduced in comparison to control (S1 Fig). As expected, radiation and combined thermoradiotherapy significantly inhibited A549 cell proliferation 48 and 72 h after irradiation but there was no significant difference in cell proliferation between radiation/hyperthermia alone and combined thermoradiotherapy treatment, e.g. no additive effect of combined treatment (S1 Fig).

### Different settings of hyperthermia treatment affect thermoradiotherapy outcome

**Fig 3:**
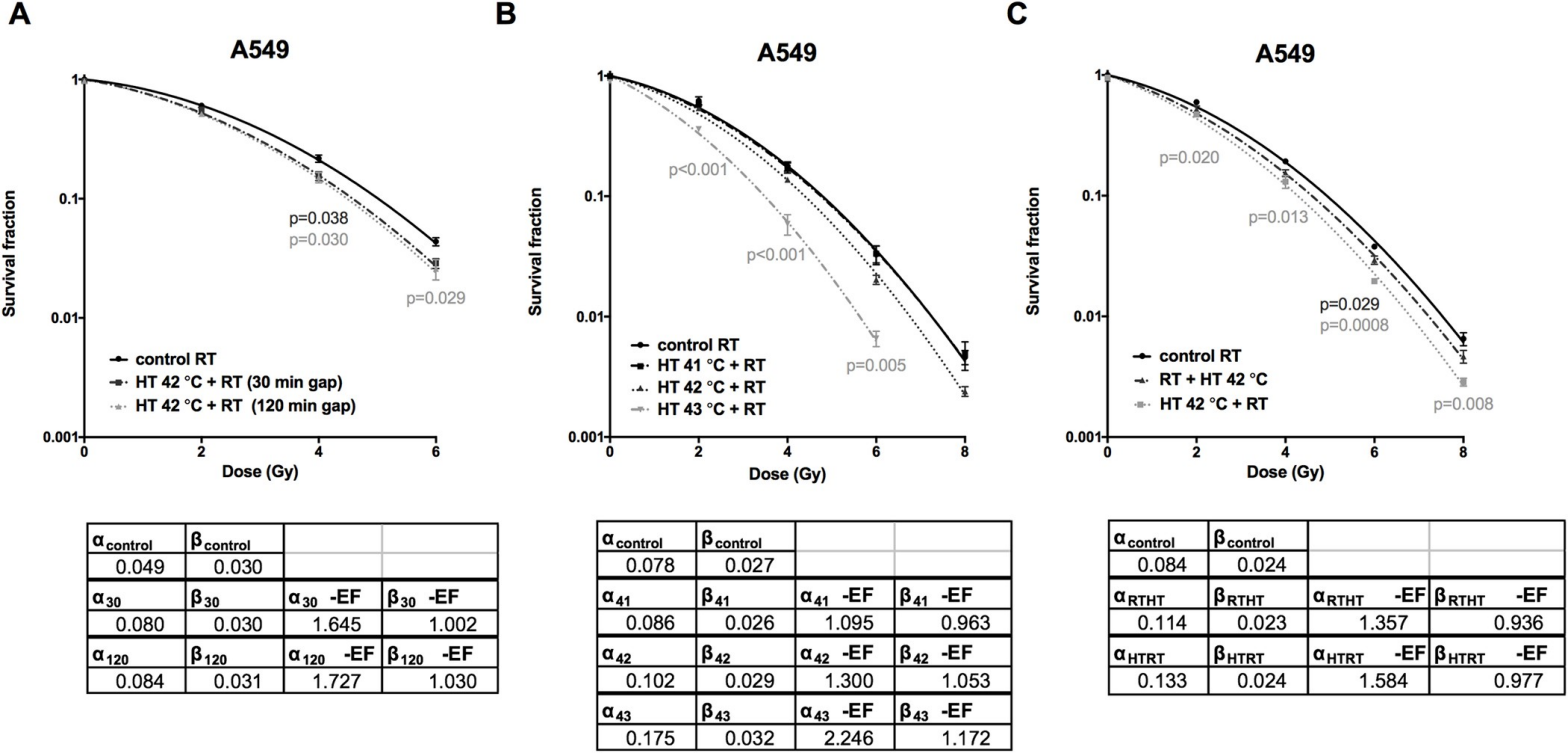
Different conditions of hyperthermia pre-treatment affect radiation-sensitivity of A549 cells. Effect of the time gap between hyperthermia and radiation on clonogenic cell survival (A). Influence of the hyperthermia temperature of 41, 42 and 43°C in combination with radiation on A549 clonogenic cell survival (B). Treatment with hyperthermia (42°C, 1 h) first versus radiation first affects clonogenic cell survival of A549 cells (C). One-way ANOVA with Dunnett’s multiple comparison test. Mean of at least three independent experiments ±SEM is shown.

To further characterize the optimal settings for hyperthermia pre-treatment *in vitro*, we tested different setup conditions of hyperthermia in A549 cell line and measured clonogenic cell survival. First, we tested if the time gap between hyperthermia and radiation plays a role in the treatment outcome. Treatment with hyperthermia prior to irradiation significantly decreased the clonogenicity of human lung adenocarcinoma cell line at 4 and 6 Gy, regardless of whether the cells were irradiated 30 or 120 min after hyperthermia treatment (Fig 3A). The EF of the LQ parameters reflect this observation; they are very similar for the 30 and for the 120 min hyperthermia treatment. Next, we tested the three clinically relevant temperatures (41, 42 and 43°C, 1 h) in combination with radiation (Fig 3B). In this setting, pre-treatment with 41°C did not show any effect (EF of the LQ parameters approximately 1), while pre-treating with 43°C significantly reduced clonogenic cell survival in response to radiation. Correspondingly, the α-EF for the 43°C treatment was the largest one (2.2) found for the A549 cell line in this work. Subsequently, we tested the order of the treatments. Treatment with hyperthermia before radiation was more efficiently inhibiting A549 cancer cell clonogenicity than treatment with radiation first and then hyperthermia when compared to cells treated with radiation alone (Fig 3C). As before, this is reflected in an increased α-EF for the case where hyperthermia was applied before radiation.

### DNA damage and apoptosis/necrosis after thermoradiotherapy treatment

**Fig 4:**
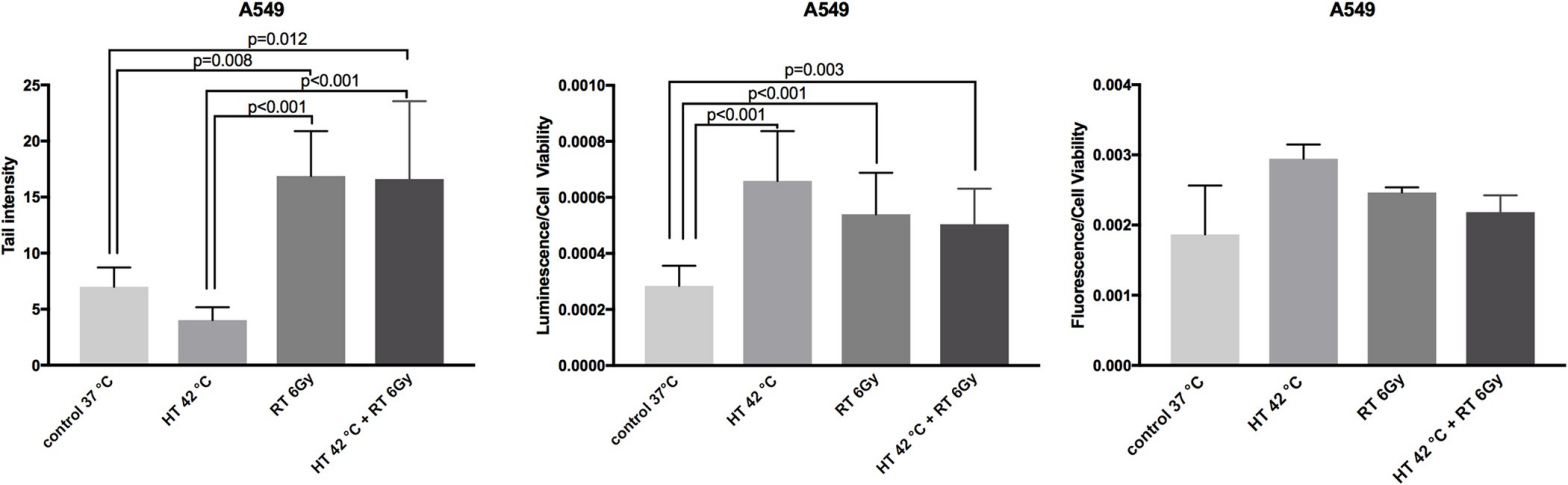
Analysis of DNA damage and apoptosis/necrosis in hyperthermia, radiation and combined thermoradiotherapy-treated A549 cells. DNA damage (tail intensity analyzed by Comet assay) in A549 cells (A). Apoptosis (B) and necrosis (C) in A549 cells 48 h after treatment. Luminescent/fluorescent signal intensity values were normalized by the number of cells (coupled luminescence assay) in each well. Mixed model ANOVA with Tukey’s multiple comparison test. Mean of three independent experiments ±SEM is shown.

As next step, we investigated the level of DNA damage in single and combined thermoradiotherapy-treated A549 cells. The alkaline Comet assay was used as it can detect and quantify effects of lower doses of radiation, more clinically relevant for our work. Radiation and combined thermoradiotherapy significantly induced increase in tail lenght in comparison to control and hyperthermia alone-treated cells, however there was no significant difference between radiation and combined thermoradiotherapy-treated cells (Fig 4A). Additionally, we tested induction of apoptosis and necrosis levels by single and combined treatment 48 hours after completion of the treatment. All three treatment-modalities (hyperthermia, radiation and thermoradiotherapy) significantly induced apoptosis but not necrosis in comparison to control cells, however we did not observe additional enhancement when comparing thermoradiotherapy to single hyperthermia/radiation treatment (Fig 4B and 4C).

### Levels of HSP70 and Rad51 in hyperthermia-radiosensitized and -resistant cell lines

**Fig 5:**
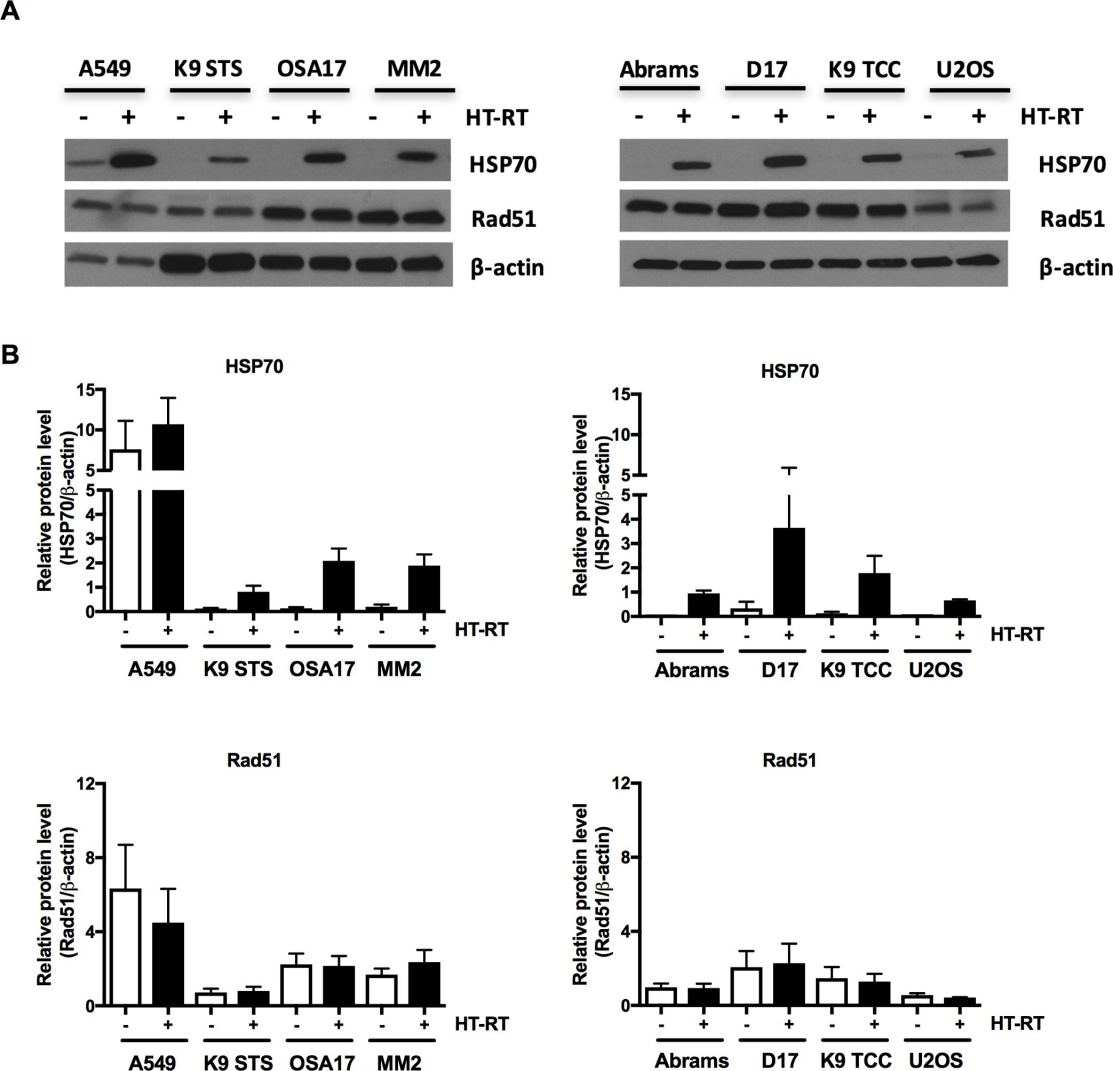
Levels of HSP70 and Rad51 in control and thermoradiotherapy-treated human and canine cancer cell lines. Cell lysates analyzed by immunoblotting for HSP70 and Rad51 and β-actin in control (-) and thermoradiotherapy (+; (42°C, heat-up plus 1 h) followed by ionizing radiation (6 Gy))-treated cells (A). Representative experiment of three experiments performed independently is shown. Quantification of HSP70 and Rad51 immunoblot signal normalized to β-actin (B). Mean of three independent experiments ±SEM is shown.

To study the role of HSP70 in hyperthermia-treatment sensitivity, we measured the baseline and thermoradiotherapy-induced HSP70 levels in eight cell lines shown in Fig 2. Interestingly, only A549 had high baseline levels of HSP70, which were further induced upon thermoradiotherapy treatment (Fig 5A and 5B). The remaining seven cell lines had very low or not detectable HSP70 levels in comparison to A549 in untreated cells with strong induction upon thermoradiotherapy treatment. We have also analyzed levels of Rad51 protein upon thermoradiotherapy treatment which were unaffected in all cell lines tested (Fig 5A and 5B).

### Effect of HSP70 Knockdown on thermoradiotherapy outcome

**Fig 6:**
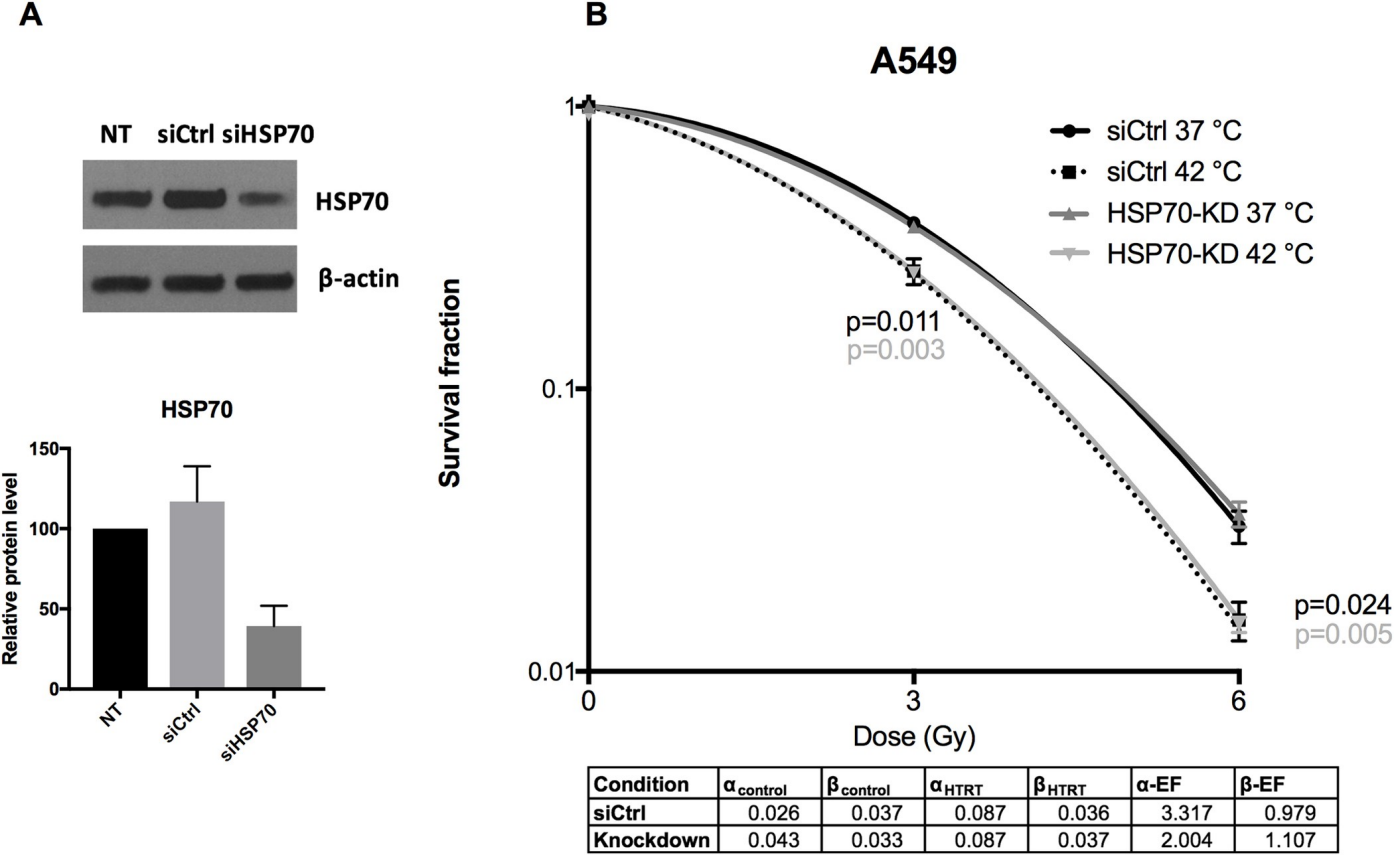
HSP70 knockdown in A549 cells. Efficiency of HSP70 downregulation 48 h after transfection in A549 cells; immunoblot (upper panel) and relative quantification of HSP70 normalized to β-actin (lower panel) and normalized to non-transfected cells, mean of three independent experiments ±SEM is shown (A). Effect of HSP70 knockdown on clonogenic cell survival of A549 cells treated with radiation or combined thermoradiotherapy (B). A control siRNA (siCtrl) was used versus HSP70-KD cells. The corresponding LQ parameters along with their enhancement factors are given in a table (lower panel). Unpaired t-test.

To further investigate the role of heat shock proteins in thermoradiotherapy-treatment response, we generated transient knockdown of HSP70 in A549 cells. In contrary to HSP70, HSP90 was not induced by hyperthermia alone in A549 cells (S2 Fig), therefore we generated knockdown of HSP70 to study its role in thermoradiosensitization. HSP70 knockdown cells and siControl-transfected cells were treated with radiotherapy alone or combined thermoradiotherapy 48 h after transfection and plated for analysis of clonogenic cell survival.Knockdown efficiency was confirmed by immunoblotting (Fig 6A). Interestingly, transient knockdown of HSP70 did not result in further decrease of clonogenic cell survival in response to radiotherapy alone and thermoradiotherapy when compared to control cells (Fig 6B). The radiosensitizing effect of hyperthermia yields and enhancement for the α parameter (2.0, 3.3) while the β parameter largely remains unchanged (β-EF ≈ 1).

## Discussion

Hyperthermia is mostly an experimental treatment modality used to sensitize tumors to radiotherapy in human and veterinary oncology, only a small number of centers is applying HT-RT in clinical routine for some specific indications (bladder, breast wall recurrence of mammary carcinoma) [12, 16]. In order to exclude the effects of the tumor microenvironment and focus on cancer cell response alone, we deliberately tested the effect of thermoradiotherapy treatment on a panel of human as well as canine cancer cell lines in vitro. Human cell lines A549 and U2OS were used for comparison, A549 as a ‘positive’ control cell line for hyperthermia as its sensitivity to it was shown before [17]. Interestingly, the majority of the canine cancer cell lines were not sensitized by hyperthermia. The effect of hyperthermia on sensitization of tumor is observed mainly *in vivo*, indicating that the tumor microenvironment is necessary for the radiosensitizing effect of hyperthermia. Increased tumor perfusion and decreased hypoxia are the major contributors to increased radiosensitization *in vivo*. However, hyperthermia can sensitize cells growing *in vitro* by potentially affecting DNA repair and *in vivo* due to an additional effect on the microenvironment. Additionally, immunomodulatory effects of HSPs induced upon hyperthermia treatment also contribute to treatment response [7]. None of these factors is present in the *in vitro* setting, when cancer cell lines are grown in 2D cultures. Not surprisingly, we observed radiosensitizing effects of hyperthermia in only two out of eight cell lines tested. The majority of sarcoma/osteosarcoma cell lines (the tumor type commonly treated with hyperthermia in the veterinary clinic) was resistant to hyperthermia treatment *in vitro*, e.g. on the pure cellular level [11]. We chose a treatment at 42°C for a time period of 1 hour to screen different cell lines for their thermoradiotherapy-sensitivity as this is the most clinically relevant temperature, and as it does not have a strong cytotoxic effect on cancer cells alone. A previous study showed various degrees of cell cytotoxicity at 42°C, van den Tempel et al. found decreasing cell survival in response to thermoradiotherapy in the temperatures above 41°C and the degree of decrease was dependent on the cell line and duration of the hyperthermia pre-treatment in different human cancer cell lines: BLM, HeLa, FaDu and VH10-SV40 [18]. As predicted, we observed no radiosensitization at 41°C and the strongest decrease in clonogenic survival at 43°C. In our study, the time gap between hyperthermia treatment and radiation (30 min versus 120 min) appeared to have no effect on the cell survival. This is in line with previous reports, where the radiosensitizing effect of hyperthermia was maintained even for up to several hours after hyperthermia treatment in cervical carcinoma cell lines [19]. It is, however, not in line with reports where the best effect was observed or predicted with a short time-gap in *vitro* [20, 21], and *in vivo* [22, 23]. Treatment with hyperthermia before radiation was more effective than the opposite setup, which has also recently been shown in fibrosarcoma tumor xenografts [24]. This setting is especially valid *in vivo*, as pre-treatment with hyperthermia can lead to increased perfusion, followed by reoxygenation and resulting radiosensitization. Interestingly, we could observe it also *in vitro*, which suggests that there are intracellular pathways being activated in response to hyperthermia that render the cells more radiosensitive. One of the radiosensitizing effects of hyperthermia could be potentially attributed to decrease in DNA damage repair after thermoradiotherapy treatment and/or increased DNA damage. However, we could not detect increased DNA damage as measured by Comet assay in response to hyperthermia alone. Possibly, the clinically-relevant dose of 6Gy is already inducing the high amount DNA damage (saturated signal) and further increase by hyperthermia cannot be measured in this setting. As predicted, radiation alone and thermoradiotherapy induced increased formation of DNA breaks in A549 cells but there was no additional increase in DNA damage upon thermoradiotherapy treatment in comparison to radiation alone. It could also suggest that thermoradiotherapy treatment does not increase the number of inflicted DNA damage but rather could affect the DNA repair pathway [25]. Indeed, it has been shown recently, that hyperthermia-treated cells and tumors have decreased levels of BRCA2 protein [18, 26]. Moreover, it has been shown that mild hyperthermia inhibits homologous recombination and induces BRCA2 degradation [27].

Levels of HSP70 protein are induced in many types of malignancies. Our observation showed that only A549 had high baseline levels of HSP70 in comparison to other cell lines tested, another hyperthermia-sensitized cell line Abrams had low baseline levels. However, all cell lines showed strong induction upon thermoradiotherapy treatment, which suggests that the baseline and induced levels of HSP70 may not be sufficient to predict the response to thermoradiotherapy treatment *in vitro*. Moreover, we analyzed levels of Rad51 protein in cells exposed to thermoradiotherapy. It was previously shown that radiation induced Rad51 foci are transiently inhibited by hyperthermia and localization and size of Rad51 foci was affected by the thermal dose [18, 28]. However, we did not observe differences in Rad51 protein levels analyzed by immunoblotting in any of the cell lines analyzed.

We observed no beneficial effect of knockdown of HSP70 on (thermo)radiosensitivity of A549 cells. Murakami et al., showed that mouse mammary carcinoma cell line 4T1 with low cytosolic and membrane-bound HSP70 was significantly more radiation-sensitive than control cells [29]. Moreover, human colon adenocarcinoma CX^+^ with a stable high membrane-bound HSP70 (mHSP70) was significantly more radiation-resistant that CX^-^ with stable low levels of mHSP70; interestingly, CX^+^ and CX^-^ have equal levels of cytosolic HSP70, which suggests that mHSP70 and not cytosolic HSP70 is responsible for radiation sensitivity [29]. In the same study, no significant differences in radiation sensitivity in H1339 and EPLC-272H lung carcinoma cells with heat shock factor-1 (HSF-1) knockdown versus control were observed, which have significant differences in cytosolic but not mHSP70 levels [29]. Since in our study we only analyzed the cytosolic levels of HSP70 in A549 upon knockdown, which were decreased in comparison to control cells, it might explain the lack of (thermo)radiosensitization of A549 when only cytosolic HSP70 levels are reduced. Furthermore, Vriend et al. and Krawczyk et al. previously showed in their work that inhibiting HSP90 increased radiation sensitivity of various cell lines [27, 30]. We chose to focus on HSP70 as we observed it was modulated by heat in our cell lines.

Regarding the applied method for hyperthermia, we used a direct heat CO_2_ regular incubator, since it shows a similar temperature profile to the hyperthermia applicator used for clinical treatments. Our aim in this study was not to achieve the fastest heat-up possible, but rather to put effort into establishing conditions that are similar to what we have during patient treatment. Our measurement rules out an excessively slow heat-up phase in which the target temperature would never have been reached. Additionally, we want to point out that our measurements include potential effects of evaporative cooling since we measured the liquid directly. Moreover, we considered the potential influence of thermotolerance, a phenomenon where cells continue to function normally after pre-exposure to high temperature, and which is attributed to increased HSPs [31–34]. We consider our method of heating not to induce thermotolerance, however this concern should be further investigated as it could reduce the sensitivity of cells towards radiation.

In conclusion, we showed that hyperthermia has limited potency to radiosensitize canine cancer cells grown in a 2D cell culture setting used in our study. Tumor cell-type, temperature and order of the treatment play are relevant factors in inducing radiosensitization by hyperthermia. Moreover, the majority of the cell lines used in our study are of mesenchymal origin, which could be be the reason of their intrinsic radioresistance. We did not observe increased DNA damage and apoptosis/necrosis upon combined treatment in comparison to single radiotherapy/hyperthermia treatment. We chose a 48 hours time point as previous studies showed that apoptosis and necrosis are induced by radiation and hyperthermia from that time [35, 36]. However, the changes might be observed at later time points, not analyzed here. Moreover, cytosolic levels of HSP70 alone appear not to play critical role in the hyperthermia-radiosensitization of lung adenocarcinoma cell line A549. Possibly, the membrane form of HSP70 is more critically involved in the radiosensitization mechanism. Further studies in different cell lines are required to analyze this issue.

## Supporting information

S1 Fig Proliferation of A549 cells in response to single and combined thermoradiotherapy analyzed at 24, 48 and 72 h after treatment. Mixed model ANOVA with Tukey’s multiple comparison test. Mean of three independent experiments ±SEM is shown.
(TIF)

S2 Fig Levels of HSP70 and HSP90 in response to heat in A549 cells. Cell lysates were collected before, during heat-up phase, during hyperthermia treatment (42°C, 1h) and at indicated time-point after treatment. Representative experiment of three experiments performed independently is shown.
(TIF)
