# Correction: Cell line-specific efficacy of thermoradiotherapy in human and canine cancer cells *in vitro*

**DOI:** 10.1371/journal.pone.0222142

**Published:** 2019-08-30

**Authors:** The PLOS ONE Staff

The fifth author’s initials are indexed incorrectly in PubMed. The publisher apologizes for this error. The correct initials are: Rohrer Bley C. The correct citation is: Nytko KJ, Thumser-Henner P, Weyland MS, Scheidegger S, Rohrer Bley C (2019) Cell line-specific efficacy of thermoradiotherapy in human and canine cancer cells *in vitro*. PLoS ONE 14(5): e0216744. https://doi.org/10.1371/journal.pone.0216744
