# Supplementary figures and images for: Cell line-specific efficacy of thermoradiotherapy in human and canine cancer cells in vitro
Source: PLoS One. 2019 May 15;14(5):e0216744. doi: 10.1371/journal.pone.0216744 (PMC6519812; doi:10.1371/journal.pone.0216744)

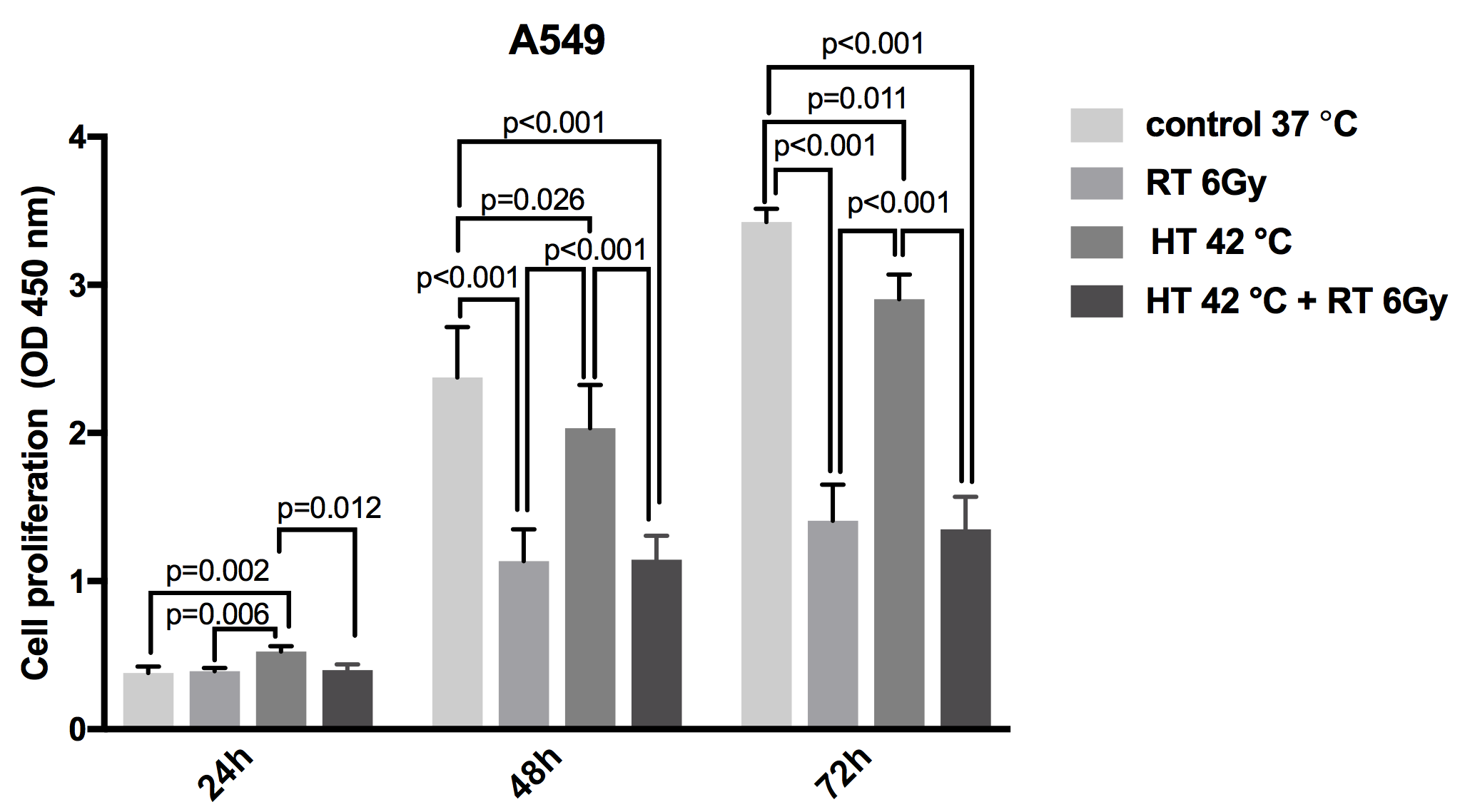

Supplement: S1 Fig — Mixed model ANOVA with Tukey’s multiple comparison test. Mean of three independent experiments ±SEM is shown. (TIF) [file pone.0216744.s001.tif]

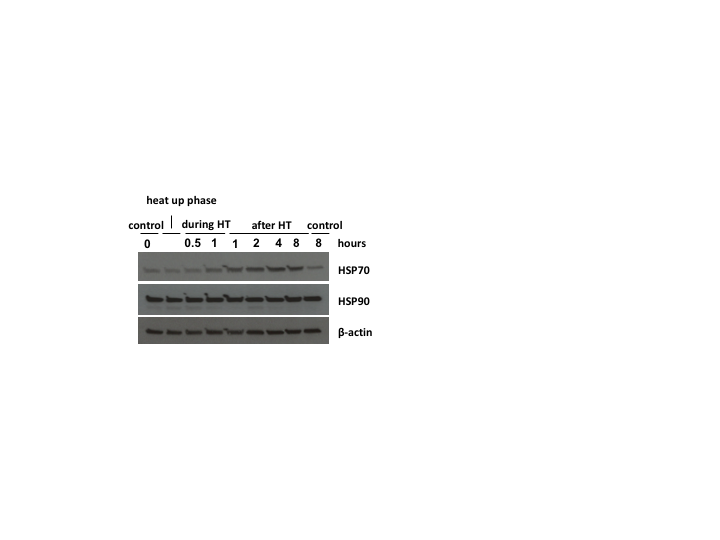

Supplement: S2 Fig — Cell lysates were collected before, during heat-up phase, during hyperthermia treatment (42°C, 1h) and at indicated time-point after treatment. Representative experiment of three experiments performed independently is shown. (TIF) [file pone.0216744.s002.tif]
